# A Phenological Timetable of Oak Growth under Experimental Drought and Air Warming

**DOI:** 10.1371/journal.pone.0089724

**Published:** 2014-02-24

**Authors:** Thomas M. Kuster, Matthias Dobbertin, Madeleine S. Günthardt-Goerg, Marcus Schaub, Matthias Arend

**Affiliations:** 1 Forest Dynamics, Swiss Federal Research Institute WSL, Birmensdorf, Switzerland; 2 Institute of Terrestrial Ecosystems ITES, ETH Zürich, Zürich, Switzerland; 3 Soil and Ecosystem Ecology Group, University of Manchester, Manchester, United Kingdom; Lakehead University, Canada

## Abstract

Climate change is expected to increase temperature and decrease summer precipitation in Central Europe. Little is known about how warming and drought will affect phenological patterns of oaks, which are considered to possess excellent adaptability to these climatic changes. Here, we investigated bud burst and intra-annual shoot growth of *Quercus robur*, *Q. petraea* and *Q. pubescens* grown on two different forest soils and exposed to air warming and drought. Phenological development was assessed over the course of three growing seasons. Warming advanced bud burst by 1–3 days °C^−1^ and led to an earlier start of intra-annual shoot growth. Despite this phenological shift, total time span of annual growth and shoot biomass were not affected. Drought changed the frequency and intensity of intra-annual shoot growth and advanced bud burst in the subsequent spring of a severe summer drought by 1–2 days. After re-wetting, shoot growth recovered within a few days, demonstrating the superior drought tolerance of this tree genus. Our findings show that phenological patterns of oaks are modified by warming and drought but also suggest that ontogenetic factors and/or limitations of water and nutrients counteract warming effects on the biomass and the entire span of annual shoot growth.

## Introduction

The growing season of deciduous trees is commonly defined as the time between bud burst and autumnal leaf senescence. More specifically, it refers to the period of annual shoot, stem and root growth. In spring, bud burst is driven by the degree of preceding winter chilling, the increasing length of the photoperiod and by rising temperatures [1], [2]. Winter chilling and short photoperiod are known to delay early bud burst and prevent frost damage, whereas rising temperatures promote bud burst [3], [4]. Similarly, the ontogenetically fixed cessation of tree growth and autumnal down-regulation of physiological functions are mainly controlled by photoperiod and temperature [5], [6], [7], [8], [9], [10], [11]. Based on the IPCC scenario A2, spring (+2.2 to 4.2°C) and autumn temperatures (+2.4 to 5.0°C) are both predicted to increase in Central Europe throughout the 21^st^ century [12], [13]. Therefore, through climate change, extended annual growing seasons may be expected due to both earlier bud burst and later cessation of growth [7], [14], [15]. Climate change is also predicted to reduce summer precipitation in Central Europe between 21 and 28% [12], [13]. Consequently, interactions between rising temperature and reduced water availability may modify growing season’s extensions.

Oaks are regarded as popular tree species in future forestry. They are considered as to be tolerant against drought and heat due to their xeromorphic adaptations in leaf and wood structure and root growth [16], [17], [18], [19]. Among several deciduous tree species, phenological parameters of oaks have been described to be most flexible to changes in temperature [20]. Whereas positive effects of warming on bud burst in trees including oaks are known [3], the consequences of increased temperatures on intra-annual shoot growth and carbon sequestration in oaks still require further investigation. In oaks, intra-annual shoot growth is of particular importance as this tree genus is able to flush several times during a growing season. In general, an overall positive response of warming on plant growth might be expected due to accelerated plant metabolism and increased soil nutrient turnover rates [21], [22]. Thus, warming and its effects on oak trees might be advantageous for future sequestration of atmospheric carbon [2], [23].

Not only warming but also decreased precipitation is predicted to influence oaks as a consequence of climate change. Reduced water availability has not been found to affect leaf unfolding and senescence in *Quercus robur* and *Q. petraea* seedlings [24]. However, drought has been reported to affect intra-annual shoot growth of oaks by reducing the number of flushes and delaying the onset of a 2^nd^ flush later in the growing season when water availability becomes more limited than in spring [25]. Only little is known about the resilience of oaks after several drought periods or how increasing temperature affects this relationship.

Based on the literature presented above, we hypothesised that (i) an increase in air temperature promotes bud burst and increases shoot growth duration leading to enhanced biomass production of oaks, but (ii) drought will counteract these effects by reducing shoot growth duration and therefore biomass. To test these hypotheses, including the resilience after re-watering and the interaction between temperature and drought, we conducted a fully-factorial model ecosystem experiment in which various provenances of *Q. robur*, *Q. petraea* and *Q. pubescens* were grown on two different forest soils and subjected to drought and air warming. Bud burst and intra-annual shoot growth were assessed over the course of three growing seasons to consider potential ontogenetic effects on these phenological traits.

## Materials and Methods

### Study Site & Experimental Design

The study was part of the multidisciplinary experimental setup “Querco” [26]. The experimental design of the model ecosystem facility (MODOEK) located at the Swiss Federal Research Institute WSL, Birmensdorf, Switzerland (47° 21′48′’ N, 8° 27′ 23″ E, 545 m a.s.l.) is described in detail in [27]. Briefly, the facility consists of 16 hexagonal chambers of 3 m height and a surface area of 6 m^2^ each. Each chamber is split below ground into two 1.5 m deep concrete-walled lysimeters. In spring 2005, the lysimeters were filled with a 0.5 m drainage packing composed of 3 layers of pure quartz gravel of decreasing grain size (from bottom to top) and a 1 m soil layer on top of this drainage layer. In one lysimeter of each chamber the soil consisted of two layers of acidic loamy sand taken from a Haplic Alisol (pH 4.0, subsoil 0.15–1.00 m, topsoil 0–0.15 m), while in the other lysimeter it consisted of a single layer of calcareous sandy loam taken from a Calcaric Fluvisol (pH 6.9). Further soil properties, including nutrient concentrations of the two soils, are presented in Table 1, [27] and [28].

**Table 1:** Physical and chemical soil properties.

	Acidic Soil		Calcareous Soil	
Depth	0–0.15 m	0.15–1 m	0–0.15 m	0.15–1 m
Texture (% sand,silt, clay)	85, 10, 5	87, 8, 5	71, 18, 12	71, 18, 12
pH (0.01 M CaCl_2_)	3.93	4.00	6.85	6.89
C_tot_ (%)	2.06	0.48	2.20	1.85
N_tot_ (%)	0.11	0.03	0.07	0.05
N_av_ (mg kg^−1^)	4.10	3.30	5.91	4.93
P_tot_ (mg kg^−1^)	524.46	469.43	422.81	325.54
P_av_ (mg kg^−1^)	3.91	4.78	3.08	2.23
Ca_exch._ (mg kg^−1^)	364.07	142.21	1798.63	1544.88
Mg_exch._ (mg kg^−1^)	25.40	9.48	29.38	18.12
K_exch._ (mg kg^−1^)	30.69	18.98	31.26	21.50
Mn_exch._ (mg kg^−1^)	45.79	18.63	1.46	1.43

Soil properties of the acidic and calcareous soils were measured at different depths (topsoil: 0–0.15 m, subsoil: 0.15–1 m) at the end of the experiment in autumn 2009. Table adapted from [27] and [28].

In spring 2006, two-year-old saplings of *Q. robur*, *Q. petraea* and *Q. pubescens* were randomly planted and grown with sufficient water supply at ambient air temperature [29]. Each species was represented by four different provenances from Switzerland and in one case (*Q. pubescens*) from Northern Italy [17]. Climatic conditions at the 12 oak stands, where the acorns were harvested in 2003, were different for all provenances [19]. Each provenance was represented by two saplings on each lysimeter (statistical unit = oak tree, *n* = 8), leading to a total number of 768 trees (4 climatic treatments, 2 soils, 12 provenances nested in 3 species, 8 replications).

From February 2007 to October 2009, each chamber was subjected to one of the following four treatments: air warming (AW), drought (D), the combination of air warming and drought (AWD) and control (CO). Each treatment was replicated four times and statistically arranged in a Latin square design. In the air-warming treatment, day-time temperatures were increased by 1–2°C in relation to the control by reducing the opening of the side-walls (Table 2, [27]). As temperatures in forest stands are generally lower than in an open field [30], the applied warming treatment came close to a moderate temperature increase in Central Europe throughout the 21^st^ century based on the IPCC scenario A2 [12], [13].

**Table 2:** Air temperature and drought stress during the experimental period from 2006 to 2009.

		ambient air temperature1)	effect of warming on air temperature2)	drought3)
2006	April	+0.0°C	none	none
	May to September	+0.9°C	none	none
2007	April	+5.1°C	not available	none
	May to September	−0.8°C	+1.4°C	weak
2008	April	−0.9°C	+1.2°C	none
	May to September	+0.1°C	+1.3°C	strong
2009	April	+3.2°C	+2.0°C	weak
	May to September	+1.1°C	+1.1°C	very strong

Ambient air temperature in relation to the long term mean (1981–2010), effect of warming on the daytime air temperature (8∶00–18∶00 h, UTC+1) and development of the drought stress from year-to-year.

1) difference to the norm ambient air temperatures (1981–2010) at the nearby WMO weather station Zürich/Fluntern, data provided by SwissMeteo.

2) in relation to control, see [27] for further details.

3) based on drought effects on soil water concentration [27], predawn leaf water potential [34] and foliage injury [17].

In the drought treatment, irrigation was discontinued for several consecutive weeks during the growing seasons while it was continued without interruption in the control. Irrigation was reduced by 60% in 2007 and by 43% in 2008 and 2009 in relation to the long-term mean precipitation during the growing season at the experimental site (April to October, 1961–2009). The drought stress increased from 2007 to 2009 due to longer intervals without irrigation and the developing canopies from year-to-year leading to increased water consumption of the trees [27]. After the first drought treatment in 2007, which had only little effects on intra-annual shoot growth, longer intervals without irrigation were applied in 2008 (5 to 10 weeks) and in 2009 (6 to 16 weeks) to increase the drought intensity and stimulate a treatment effect. In 2008 and 2009, the drought treatment was not stopped before soil moisture reached a threshold value of 0.05 m^3^ m^−3^ for several days [27]. At the end of each drought period, intensive irrigation was applied for a few days, simulating heavy rainfall, according to the increasing probability of extreme precipitation due to global warming. Rainfall was excluded from the chambers in all treatments during the growing seasons, while the roof tops were left open during dormant seasons to allow for natural irrigation.

### Phenological Observations

**Figure 1:**
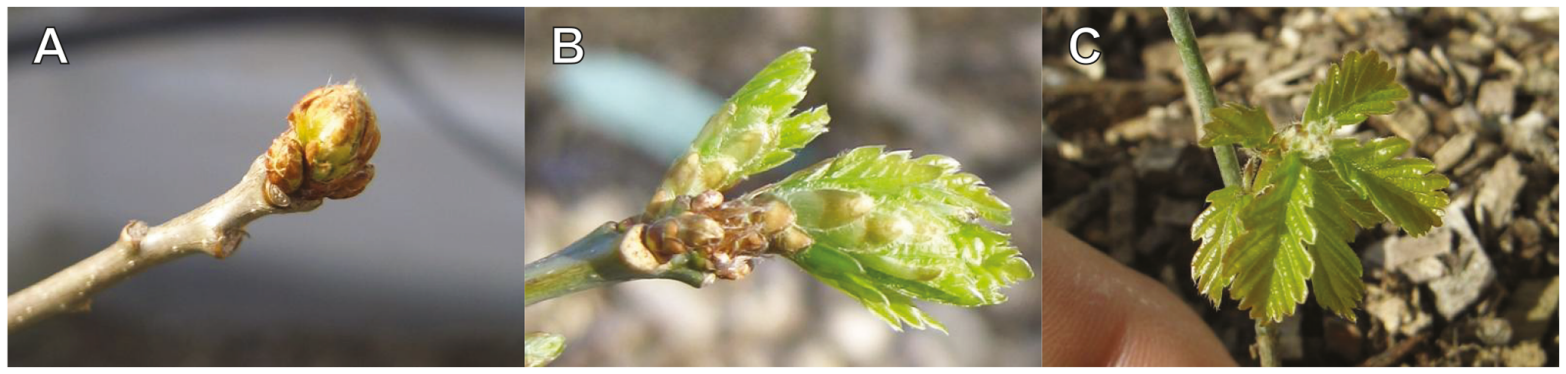
Examples of the phenological observations assessed during spring (2007 to 2009). A: greening of buds; B: bud burst; and C: leaf unfolding. The day of the year when 50% of a tree’s buds/leaves were green, burst or unfolded was used for statistical analysis and data presentation (Table 3, Figs. 2, 3, S1 and S2).

During spring in 2007, 2008 and 2009, all oak saplings were assessed bi-weekly for phenological development (greening of the buds, bud burst and leaf unfolding, see Fig. 1). In 2007, each bud was separately evaluated for phenological development. In 2008 and 2009, there were too many buds to be assessed individually so, the phenological development was estimated in 10% classes. For statistical analyses of the phenological development, we used the day of the year (DOY) when >50% of the buds of a tree attained the respective phenological stage.

### Shoot Growth Measurements

In the spring of each growing season, the shoot closest to the tip was determined as the potential main shoot. Elongation of the main shoot was measured every fourth to seventh day until the end of August and every second to third week from September until mid October. Shoot length was measured with a ruler from the last terminal bud to the newly formed bud. As oaks flush several times per growing season, we measured the shoot elongation of each flush separately.

Stem diameter increment was monitored in 2009 with one selected *Q. robur* provenance (Tägerwilen) grown on well-watered, acidic soil using automated single point dendrometers (Zweifel Consulting, Hombrechtikon, Switzerland), as described in detail in [31]. In September 2009, all aboveground wood and foliage biomass was harvested. Dry weights of shoot (aboveground wood and foliage) were determined after drying at 65°C for several days.

### Temperature and Soil Data

**Figure 2:**
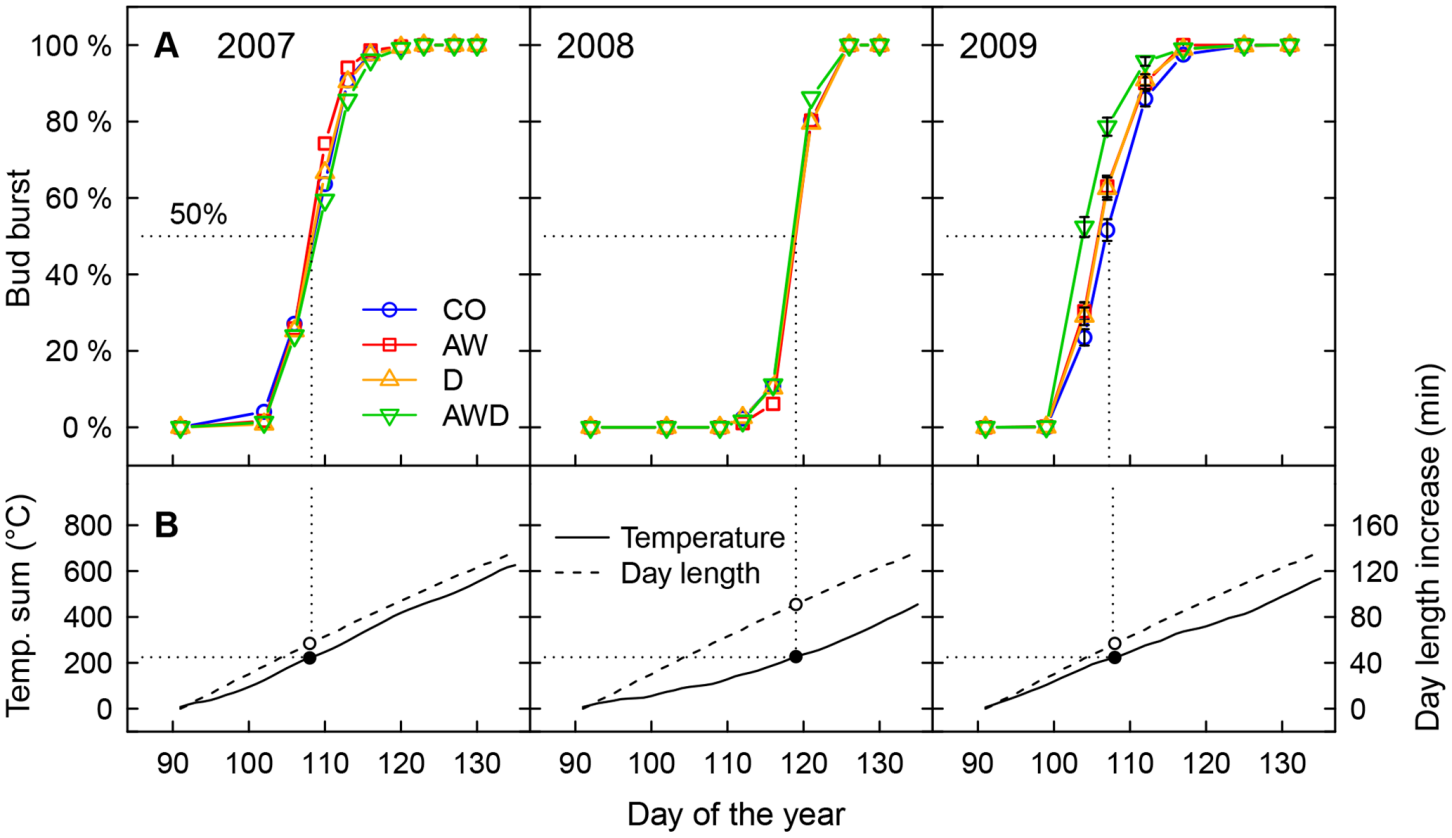
Average annual (2007 to 2009) development of bud burst. A: Relative bud burst development, separately shown for the control treatment (CO) and the air warming (AW) and drought (D) treatments and the combined treatment for air warming & drought (AWD). Data of the two soils and the 12 provenances were pooled (*n* = 192 for each treatment and year combination) as the influence of the soil on bud burst was only minor (<1 day, see text for more details) and the effect of the provenances on bud burst is shown separately in Fig. S2. For graphical reasons, error bars (± SE) are only partially shown for the 2009 development. Results of statistical analyses are given in Table 3. B: Sum of daily mean air temperature (°C) and day length increase (min) starting on day 91 (2007/2009 1^st^ April; 2008 31^st^ March). The dotted vertical line refers to the average air temperature sum (224 degree days) required for bud burst (>50% bud burst per tree). The temperature data was taken from the nearby WMO weather station Zurich Fluntern.

Air temperature in the chambers was measured every hour at a height of 120 cm with shaded EL-USB-2 data sensors (Lascar Electronics Ltd, Whiteparish, UK), with initial measurements in June 2007 [27]. As there was no temperature data available for spring 2007, we used temperature data from the nearby WMO weather station Zurich Fluntern as a substitute in Fig. 2 (data provided by MeteoSwiss).

At the end of the experiment in 2009 (26 October – 5 November) soil samples (each pooled from five soil cores of 3 cm diameter) were taken at different depths from each lysimeter (topsoil: 0–0.15 m; subsoil: 0.15–1 m). Available soil nitrogen (N_av_: NO_3_^−^+NH_4_^+^) was extracted with 1 M KCl and measured in fresh samples as described in [28]. Remaining soil samples were dried at 105°C, ground and sieved (>1 mm). For measuring available phosphate (P_av_), soil samples were extracted with 0.5 M NaHCO_3_ (Olsen-P), following [32]. The concentrations of P_av_ in the extracts were detected by spectrophotometry at 880 nm (Cary 50 UV-VIS, Varian, Palo Alto, California, USA). Exchangeable cations (Ca_exch._, Mg_ exch._, K_ exch._ and Mn_ exch._) were extracted with 0.1 M BaCl_2_ according to [32] and detected using an ICP-OES (Vista MPX, Varian, Palo Alto, California, USA). Total soil carbon (C_tot_), nitrogen (N_tot_) and phosphorous (P_tot_) were measured by means of a dry combustion analyser (CN-2000, LECO Corp., St. Joseph, Michigan, USA) and X-ray fluorescence (X-Lab 2000, Spectro, Kleve, Germany), respectively. Soil pH was measured in 0.01 M CaCl_2_.

### Statistical Analysis

For statistical analyses, we used R 2.11.1 (R Development Core Team, Vienna, AT). The data were analysed after log-transformation by ANOVA using a linear mixed-effects model accounting for the split-plot design of the experiment with two soils in each chamber (significant at *P*<0.05). The oak provenances were nested within species and compared using the mean of each provenance versus those of all other provenances (significant at *P*<0.05). Selected differences between treatments and soils were tested pair-wise using contrasts based on *t*-tests (significant at *P*<0.01). Correlation factors (*r*) were calculated based on the Pearson method (significant at *P*<0.05).

## Results

### Timing of Bud Burst

The timing of bud burst was correlated with the preceding greening of the buds (*r* = 0.807) and the subsequent unfolding of the leaves (*r* = 0.939). As the effects of the climatic treatments, soils, growing seasons and species/provenances were very similar in all three phenological stages being assessed, only the results from bud burst are presented and discussed in detail.

Bud burst was mainly triggered by air temperature, which differed significantly between the three growing seasons, but also between the climatic treatments (Table 2, [27]). In April, when the growing season for oaks starts, mean ambient air temperature was lower in 2008 (8.0°C) compared to 2007 (13.9°C) and 2009 (12.0°C). Consequently, in 2008, bud burst (>50% of the total number of a tree’s buds) of control oaks was 11 days later (DOY 119) than in 2007 and 2009 (DOY 108; Fig. 2A). Across all three growing seasons, 224 degree days (sum of mean daily temperature in °C) in April triggered >50% bud bursts per tree (Fig. 2B). We found that April temperatures were sufficient to explain patterns in bud burst and applying more complex phenological models including early spring temperature, chilling threshold [4] or day length [33] did not help explain the uncertainty left in the present study.

**Figure 3:**
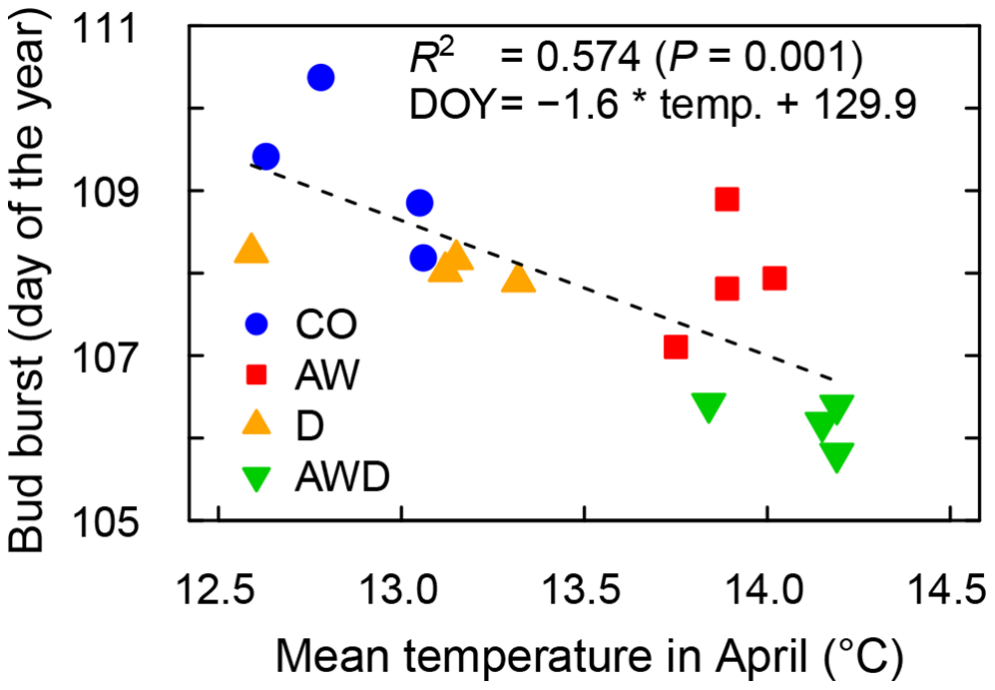
Relationship between mean April air temperature in 2009 and bud burst phenology. Air temperature (°C, 0∶00 to 23∶00, UTC+1) and the day of the year when 50% of the buds were open are separately shown for each model ecosystem chamber. Data of the two soils and the 12 provenances were pooled because air temperature was equal within a chamber. Over all treatments, the negative slope of the correlation line was significant (*R* ^2^ = 0.574, *P* = 0.001). See Fig. S1 for the non-significant relationship in 2008.

The air-warming treatment influenced bud burst only in 2009 (Table 3), in that increased air temperature (2.0°C) enhanced bud burst by 1 to 2 days (Fig. 3). During the warmer 2007 spring (Table 2), bud burst was not affected by the air-warming treatment. However, we observed a temperature effect on the unfolding of the leaves (AW x provenance: *P* = 0.046), indicating that there is nevertheless a warming effect on spring phenology. In contrast to 2007 and 2009, temperatures in April 2008 were relatively low (Table 2); upon warming, bud burst presumably occurred to rapidly to detect any differences between treatments by using a bi-weekly assessment (steep increase of bud burst development curve in Fig. 2A, Fig. S1). In summary, each 1°C temperature increase led to an earlier bud burst of 1 to 3 days regardless of the source of warming (different ambient temperature between years or climatic treatments, Figs. 2 & 3).

**Table 3:** ANOVA *F*-values of bud burst, number of flushes and duration of growth.

			bud burst			number of flushes			duration of growth		
	df1	df2	2007	2008	2009	2007	2008	2009	2007	2008	2009
AW	1	12	0.0	0.0	26.9***	1.5	3.2	0.6	0.9	0	0.8
D	1	12	1.1	0.2	22.0***	9.5**	49.9***	32.4***	12.4**	59.7***	35.5***
Soil	1	12	0.5	0.2	53.8***	53.2***	79.8***	4.9*	22.0***	97.9***	23.4***
Spec	2	9	0.2	0.1	0.1	1.5	6.4*	12.5**	2.2	8.6**	7.1*
Prov	9	264	0.8	1.0	67.0***	11.2***	11.1***	10.3***	13.1***	10.7***	14.7***
AW:D	1	12	0.6	0.3	1.1	0.1	0.0	0.3	1.4	2.2	0.8
AW:Spec	2	264	2.0	0.4	0.9	0.0	3.4*	0.0	0.6	0.6	0.3
AW:Prov	9	264	1.8(*)	1.2	1.7(*)	1.4	1.9(*)	1.1	1.6	2.4*	1.6
D:Soil	1	12	0.0	0.1	0.1	2.1	0.3	16.1**	0.1	0.1	22.7***
D:Spec	2	264	0.1	0.1	3.4*	1.3	8.9***	4.1*	0.6	0.7	2.5(*)
D:Prov	9	264	0.6	0.5	1.3	0.4	3.2***	2.9**	0.3	2.3*	1.4
Soil:Spec	2	264	0.9	0.4	3.7*	8.5***	15.3***	3.8*	7.8***	13.8***	3.5*
Soil:Prov	9	264	0.2	1.2	1.5	2.4*	1.5	1.5	1.4	3.9***	1.4
AW:D:Spec	2	264	1.5	1.0	3.3*	0.7	2.1	1.2	1.2	2.4(*)	0.6
AW:Soil:Spec	2	264	0.9	0.0	1.0	2.1	0.1	0.0	3.9*	0.5	0.0
D:Soil:Spec	2	264	0.2	0.4	0.3	0.1	1.0	5.9**	0.4	0.1	2.6(*)
AW:D:Soil:Prov	9	264	0.5	0.4	1.2	1.6	0.6	2.4*	0.6	0.6	1.0

Main effects and selected two-way interactions of drought (D, continuous *vs*. discontinuous irrigation), air warming (AW, ambient *vs*. elevated air temperature), soil (acidic *vs*. calcareous), species (Spec) and provenance (Prov, nested within species) on bud burst (day of the year when 50% of buds were burst), the number of flushes per tree and duration of shoot growth, all separately shown for each year, *n* = 8. df1: degrees of freedom in the numerator; df2: degrees of freedom in the denominator; *F*-values: 0.0 refer to values <0.05; levels of significance: (*) *P*<0.1, * *P*<0.05, ** *P*<0.01, *** *P*<0.001. Non-significant interactions are not shown.

Earlier bud burst was also observed in drought-exposed oaks in 2009, although this effect was less obvious than in oaks subjected to air warming (Table 3, Fig. 3). The combination of warming and drought triggered an even earlier bud burst. No drought effect on bud burst was found in 2007 and 2008 (Table 3, Fig. S1), possibly as the oaks were not or less affected by previous years’ drought (Table 2).

Differences in bud burst between the two soils and the tested provenances were only significant in 2009 (Table 3). Bud burst of oaks grown on acidic soil was marginally earlier compared to those grown on calcareous soil. The influence of soil on bud burst was minor (*Δ* <1 day in all treatment and provenance combinations) with no significant interactions between soil and other factors. Bud burst of the provenances *Q. robur* Magadino (Mi), *Q. petraea* Magden (M), Q. *petraea* Wädenswil (W) and *Q. pubescens* Leuk (Lk) was earlier, whereas bud burst of *Q. robur* Bonfol (B) and *Q. pubescens* Promontogno (P) was later than the mean of all provenances (S2). The variation in bud burst within species was therefore stronger than between species. Furthermore, there was no interaction between the provenances and the climatic treatments on bud burst, indicating that the different provenances responded similarly to air warming and drought.

### Number of Flushes Formed within the Season

**Figure 4:**
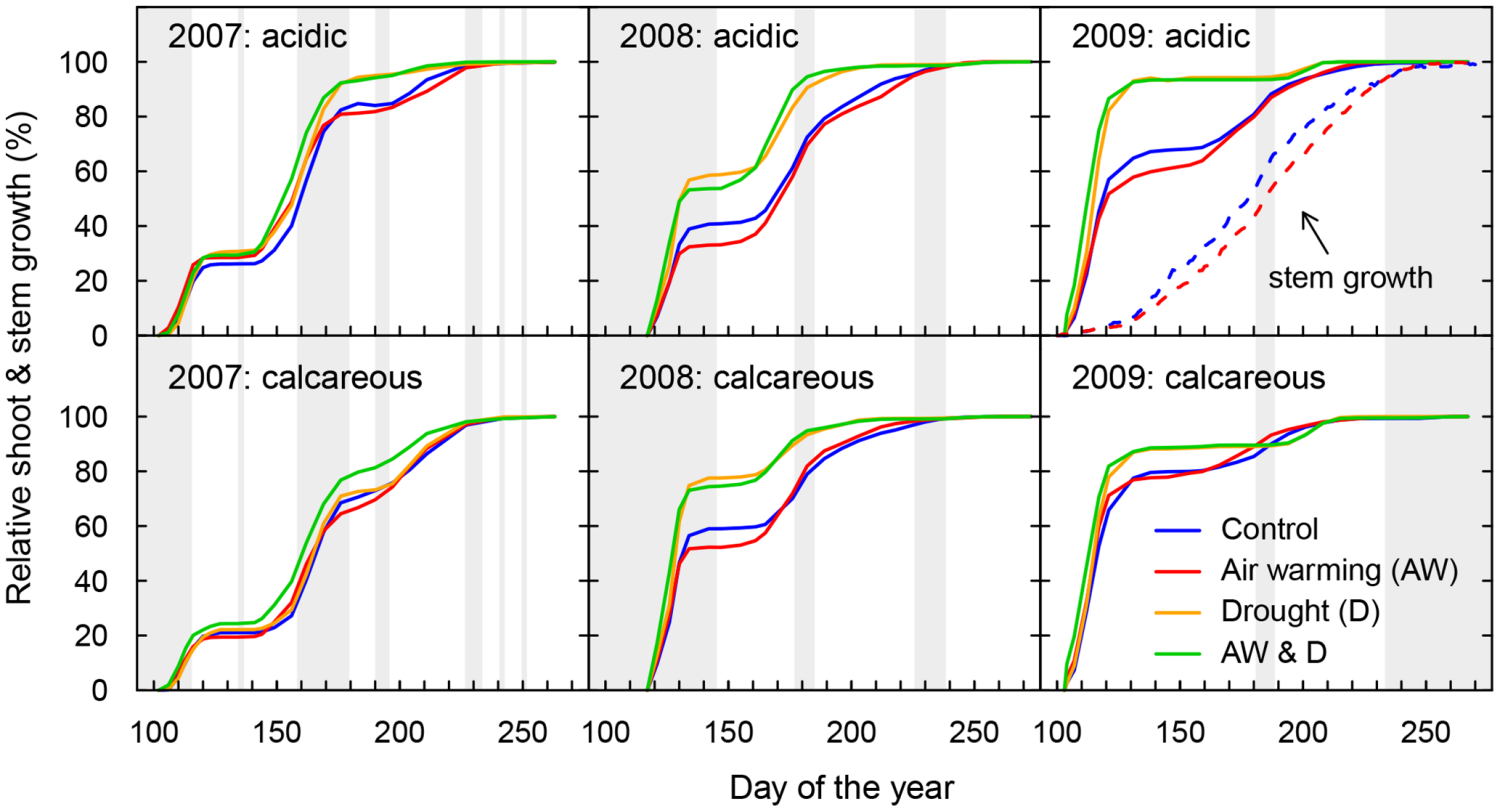
Averages of annual relative shoot growth. Growth development is separately shown for the three years (2007 to 2009), all treatments and the two soils (acidic *vs.* calcareous). Data of the 12 provenances were pooled (*n* = 96) as the effects of the species and the provenances are separately shown in Fig. S2. Additionally in 2009: example of relative stem diameter growth (*Q. robur* Tägerwilen (T)) on the acidic soil, separately shown for the control (CO) and air warming (AW) treatments, *n* = 4. The grey bars indicate periods when all chambers (including the drought treatment) were irrigated.

**Figure 5:**
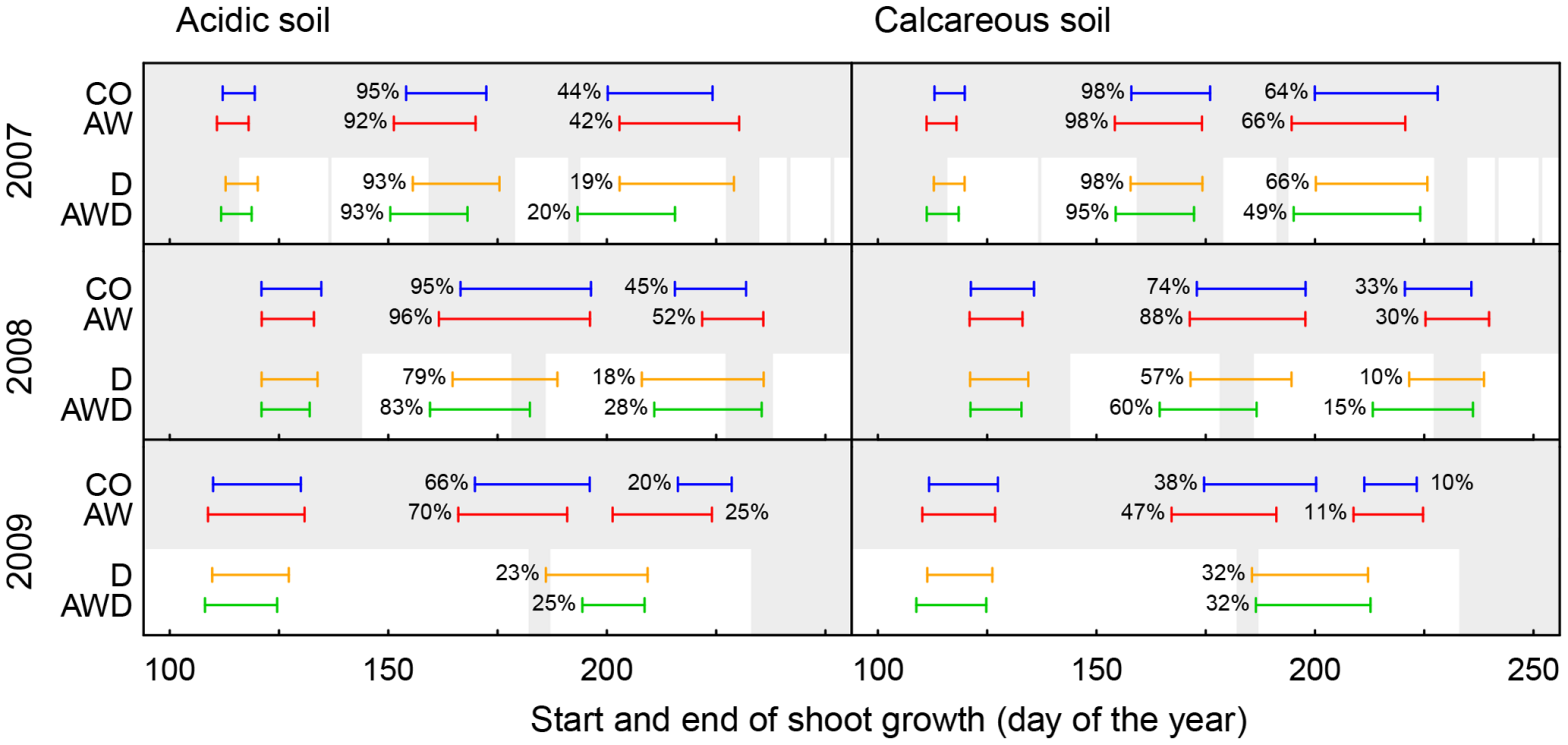
Average start, end (>95% of growth) and duration of shoot growth. Timetables are separately shown for each flush, the control treatment (CO), air warming (AW) and drought (D) treatments and the combined treatment for air warming & drought (AWD), the two soils (acidic *vs.* calcareous) and the three growing seasons (2007, 2008 and 2009). The numbers next to the bars refer to the ratio of trees with a 2^nd^ and/or 3^rd^ flush (%, 1^st^ flush always 100%). Data of the 12 provenances were pooled (*n* = 96) as the effects of the species and the provenances are separately shown in Fig. S2. Results of statistical analyses are given in Tables 3 & 5. The grey bars indicate periods when chambers were irrigated.

The mean number of flushes per tree decreased from 2.4 in 2007 (mean across all treatments, provenances and soils) to 2.1 in 2008 and to 1.5 in 2009 (Tables 3 & 4, Figs. 4 & 5), indicating an age effect on the capacity of oaks to form several flushes within a growing season. Drought significantly reduced the number of flushes by approximately 20% in 2008 and 2009, but not in 2007 when water limitation was much less pronounced due to shorter drought periods and less competition for soil water (Tables 2, 3 & 4, [27]). In contrast to drought, air warming had no influence on the number of flushes. In 2007, trees grown on the calcareous soil flushed more often than those grown on the acidic soil (*e.g.* control +10%), whereas the opposite was observed in 2008 (−15%) and 2009 (−20%).

**Table 4:** Mean number of flushes per tree 2007–2009.

	2007		2008		2009	
	acidic	calcareous	acidic	calcareous	acidic	calcareous
Control	*2.4^a^±0.1	*2.7^a^±0.1	*2.4^a^±0.0	*2.1^a^±0.1	*1.9^a^±0.1	*1.5^ab^±0.1
AW	*2.3^a^±0.1	*2.7^a^±0.1	*2.5^a^±0.1	*2.2^a^±0.1	1.9^a^±0.1	1.6^a^±0.1
D	*2.1^a^±0.1	*2.7^a^±0.0	*2.0^b^±0.1	*1.7^b^±0.1	1.2^b^±0.1	1.3^b^±0.1
AW & D	*2.1^a^±0.0	*2.5^a^±0.1	*2.1^b^±0.0	*1.8^b^±0.1	1.3^b^±0.0	1.3^b^±0.0

Air warming (AW), drought (D) and soil effects on the number of flushes per tree. Data are separately shown for each growing season (means ± SE). Data of the different provenances were pooled before statistical analyses (*n* = 8). Different letters indicate significant differences between respective treatment means on the same soil, an asterisk * indicates significant differences between acidic and calcareous soil for the same treatment (*P*<0.01).

### Shoot Elongation and Radial Stem Growth

With increasing drought stress from 2007 to 2009 (Table 2, [27]), the effect from reduced soil water availability on shoot elongation became more apparent, with the strongest effect in 2009 (Fig. 4). Mainly, the length of the 2^nd^ flush was reduced by drought. Thus, in 2009, drought-exposed oaks completed most of their total shoot elongation (>90% shoot length) within the first few weeks after bud burst when soil water availability was still sufficient. In contrast to this pattern, shoot growth from well-watered oaks, was spread over several flushes. Additionally, we observed a carry-over effect of drought on the 1^st^ flush in the following spring. Although soil water availability in spring was not different from the control at this point, shoot elongation of the 1^st^ flush of drought-exposed oaks was reduced in 2008 (−12%, *P* = 0.005) and 2009 (−13%, *P* = 0.002). Unlike longitudinal shoot growth, radial stem growth was rather continuous as demonstrated for well-watered *Q. robur* Tägerwilen trees in 2009 (Fig. 4). The start and duration of shoot growth, however, were similar and there was also no air-warming effect.

### Timing and Duration of Shoot Flushing within a Season

As expected, the start of the 1^st^ flush within a given year was strongly correlated with the preceding bud burst (*r* = 0.827). There was, however, no correlation between the 2^nd^ (*r* = 0.091) or 3^rd^ flush (*r* = 0.380) and bud burst (more correlations are shown in Table S1). In general, air warming led to an earlier onset of intra-annual flush growth, specifically in 2007, 2008 (2^nd^ flush) and 2009 (1^st^ flush and 3^rd^ flush), both under drought and well-watered conditions (Table 5, Fig. 5). For oaks exposed to air warming however, we observed no universal prolongation of the time period for flush growth as it stopped earlier compared to the flush growth for non-warmed oaks (Table 5).

**Table 5:** ANOVA *F*-values of start and duration of growth per flush.

	AW	D	Soil	AW x D	D x Soil
**Start growth**					
* 2007*					
1^st^ Flush	28.577***	1.951	0.429	0.201	3.180
2^nd^ Flush	17.433**	0.002	25.490***	0.511	0.151
3^rd^ Flush	7.129*	1.870	5.359	5.359*	1.740
*2008*					
1^st^ Flush	0.403	0.011	2.974	0.872	0.014
2^nd^ Flush	12.032**	5.068*	22.013***	0.903	0.753
3^rd^ Flush	0.187	3.413(*)	1.685	1.257	0.351
*2009*					
1^st^ Flush	29.284***	4.460(*)	18.893***	1.333	0.453
2^nd^ Flush	0.011	65.617***	0.267	4.718(*)	2.380
3^rd^ Flush	5.234(*)	–	1.561	–	–
**Duration growth**					
*2007*					
1^st^ Flush	0.106	0.013	0.347	0.012	0.525
2^nd^ Flush	0.549	1.358	0.622	0.515	2.424
3^rd^ Flush	0.682	0.004	2.214	0.070	0.192
*2008*					
1^st^ Flush	23.968***	5.139*	2.231	0.017	0.124
2^nd^ Flush	0.484	19.034***	11.032**	1.717	3.574(*)
3^rd^ Flush	0.053	8.042*	1.737	1.841	0.018
*2009*					
1^st^ Flush	1.430	19.673***	35.499***	1.784	6.738*
2^nd^ Flush	1.705	3.972(*)	3.536(*)	0.614	5.050*
3^rd^ Flush	6.778*	–	4.103	–	–

Main effects and selected two-way interactions of drought (D, continuous *vs*. discontinuous irrigation), air warming (AW, ambient *vs*. elevated air temperature) and soil (acidic *vs*. calcareous) on start and duration of growth. Data is separately shown for each growing season and each flush. Data of the provenances growing on the same half of each lysimeter were pooled before analysis (*n* = 8). *F*-values and level of significances: (*) *P*<0.1, * *P*<0.05, ** *P*<0.01, *** *P*<0.001. For all main and interaction effects: degrees of freedom in the numerator (df1) = 1 and denominator (df2) = 12. Non-significant interactions are not shown.

Drought only affected the start of the 2^nd^ flush (Table 5). In 2008, the onset of the 2^nd^ flush was advanced by the moderate drought (Fig. 5). However, in 2009 the drought was much more severe with mean predawn leaf water potentials of −1.5 and −2.9 MPa on acidic and −2.8 and −3.9 MPa on calcareous soil in D and AWD respectively (data presented in detail in [34]). Consequently, the start of the 2^nd^ flush growth was delayed by 15 days and 22 days on the calcareous and acidic soil, respectively, until the drought was interrupted by intermediate irrigation. Thereafter, growth resumed within a few days after re-watering the soil. In 2008 and 2009, drought reduced average growth duration of all flushes by 2 days. As an exception, the growth of the 3^rd^ flush in 2008 of drought-exposed oaks lasted 9 days longer compared to the well-watered trees - most likely due to re-watering during flush growth. Flush growth on acidic soil started earlier than on the calcareous soil (2^nd^ flush 2007: 4 days earlier (mean of all treatments); 2^nd^ flush 2008: 7 days; 1^st^ flush 2009: 2 days). Also, the duration of growth of some flushes was longer on the acidic than on the calcareous soil (2^nd^ flush 2008: +4 days; 1^st^ flush 2009: +3 days).

### Duration of Total Shoot Growth per Growing Season

Air warming neither increased neither the number of flushes nor the duration of single flush growth. As such, the duration of total shoot growth was not affected (Table3, Fig. S2). The duration of total shoot growth of drought-exposed oaks was shorter than that of well-watered trees (on average over all years and both soils: −18 days; Table 3, Fig. S2). This reduction in growth duration was particularly distinct on the acidic soil in 2009 when only a quarter of the trees flushed a 2^nd^ time (−35 days, Fig. 5). Differences in the duration of total shoot growth between the two soils were determined by the development of a 2^nd^ or 3^rd^ flush; the period of shoot growth was longer for oaks grown on calcareous soil than for those grown on the acidic soil in 2007, whereas it was the other way around in 2008 and 2009 (Figs. 5 & S2).

The duration of the total shoot growth differed between the provenances as well as between the three oak species (Table 3, Fig. S2). In general, *Q. robur* provenances formed more flushes per season with a longer growth period than the provenances of the other two species. Shoot growth of the *Q. pubescens* provenance Leuk (Lk) was longer, whereas the duration of growth of *Q. petraea* Wädenswil (W) and of the two *Q. pubescens* provenances Promontogno (P) and Arezzo (A) was shorter compared to the mean of all provenances.

### Shoot Biomass

After three growing seasons, total shoot biomass (aboveground wood and foliage) was significantly reduced by drought (Table 6). Shoot biomass was significantly higher on the acidic than on the calcareous soil under well-watered conditions, whereas no difference was observed between the soils under drought conditions. Air warming had no effect on shoot biomass. Further details regarding shoot biomass, including differences between species and provenances, are presented in [19].

**Table 6:** Aboveground wood and foliage biomass after three growing seasons.

	acidic	calcareous
Control	*324^a^ ±17	*258^a^ ±10
AW	*338^a^ ±26	*233^a^ ±5
D	147^b^ ±3	141^b^ ±3
AW & D	141^b^ ±7	137^b^ ±5

Air warming (AW), drought (D) and soil effects on shoot biomass (g tree^−1^) at the end of the experiment in autumn 2009. Data of the different provenances were pooled before calculating means and SE (*n* = 8). Different letters indicate significant differences between respective treatment means on the same soil (*P*<0.01). An asterisk (*) indicates significant differences between acidic and calcareous soil for the same treatment. More biomass and shoot length data are presented in [29] and [27].

## Discussion

### Warming Effects on Bud Burst and Intra-annual Shoot Growth

Advanced bud burst has shown to be a specific response of temperate tree species to increasing spring temperature [1], [2]. It has been hypothesised that climate warming might extend the growing season in temperate areas and thus increase seasonal biomass production in forest ecosystems [7], [14], [15]. In the present study, we observed advanced bud burst in oaks exposed to warming as well as an earlier start of intra-annual shoot growth. The advanced phenological development, however, was counteracted by an earlier cessation of intra-annual shoot growth. Thus, we neither observed a prolonged time period of seasonal growth nor an increased biomass production. It is therefore reasonable to assume that bud burst is triggered by environmental factors, *e.g.* temperature and photoperiod, while the duration of subsequent shoot growth is under strong ontogenetic control. Similarly, Soolanayakanahally et al. reported that bud burst of *Populus balsamifera* provenances was driven by local environmental factors whereas shoot growth duration was ontogenetically fixed as it depended on the latitude of the provenance’s origin [35]. Ontogenetic control of shoot growth duration of oaks is also in line with the sudden down-regulation of photosynthetic carbon allocation in late summer in our model ecosystem experiment, which occured independent of any obvious changes in photoperiod and temperature [34]. The treatments also did not change leaf longevity [17].

Besides ontogenetic fixed shoot development, other factors, such as nutrient availability, might also have limited growth. For example, average growth duration and shoot biomass were both lower on the calcareous than on the acidic soil, most likely due to limited availability of phosphorous (P) and manganese (Mn) [36]. Averaged over all growing seasons, treatments and provenances, leaf Mn and P concentrations of trees grown on the calcareous soil (Mn: 43 mg kg^−1^, P: 2.77 g kg^−1^) were lower than of those on the acidic soil (Mn: 2766 mg kg^−1^; P: 3.32 g kg^−1^). Leaf Mn and P concentrations of trees grown on the calcareous soil were also below the deficiency level given for oak leaves (Mn: 35–100 mg kg^−1^, P: 1.5–3 g kg^−1^, [37]). Similarly, the number of flushes, and therefore duration of shoot growth, was reduced under nutrient limiting conditions in several other studies on *Q. petraea* [38], [39], [40], [41]. We therefore conclude that warming can induce a phenological shift towards earlier bud burst and shoot growth, but, in contrast to our first hypothesis, that factors other than temperature, *e.g.* ontogenetic fixed shoot development and/or nutrient availability, limit the total time period of intra-annual shoot growth.

Advanced bud burst due to increased temperatures is in agreement with several other studies investigating temperature effects on spring phenology of European oaks [20], [24], [42], [43]. As warming of each 1°C led to a 1 to 3 day earlier onset of bud burst in our experiment, increased spring temperature up to 4°C (upper boundary of the IPCC A2 scenario [12], [13]) might advance bud burst by up to 12 days, without consideration of inter-annual variability. However, premature bud burst might be hampered by insufficient chilling, daylight or frost [44]. As demonstrated for maple trees, atmospheric CO_2_ enrichment might have no or only minimal effects on bud bust [44]. In contrast to this, we did not find any evidence for delayed growth cessation due to the applied warming in the present study; this is in line with a former study in which we failed to show delayed autumnal leaf senescence due to warming, as indicated by the loss of photosynthetic activity or chlorophyll degradation [34]. Indeed, the temperature effect on autumnal phenology has been reported to be highly variable in oak species [7], [20], [24], [43].

### Drought Effects on Bud Burst and Intra-annual Shoot Growth

Surprisingly, in 2009 the onset of bud burst for drought-exposed oaks was considerably earlier than for well-watered oaks. A direct effect of reduced water availability on bud burst can, however, be excluded as the drought in the 2009 season was not then effective during the time when buds burst and air temperatures were not increased [27]. We therefore conclude that the observed advancement of bud burst is most likely explained by a carry-over effect from the previous year’s drought. Indeed, the first flush is preformed within the bud formed during the previous season [45]. As such, a drought event during bud formation might impact the date of its outgrowth in the following season, as has been shown for evergreen oaks [46]. This conclusion is in agreement with the missing carry-over effects in the 2007 and 2008 growing seasons during which bud burst was not or less influenced by the previous year’s drought. Carry-over effects due to the previous year’s climatic conditions have also been demonstrated for the length of newly formed shoots in *Fagus sylvatica* and oaks ([47], this study), the width of tree rings in *Fagus grandifolia* and *Quercus alba* [48] and the size of early wood vessels in *Castanea sativa* [49]. While such carry-over effects on shoot length and wood growth can be simply explained by insufficient resource storage for bud formation or xylem production [50], the nature of the carry-over effect on the timing of bud burst remains so far unknown.

In support of the second hypothesis, the growth rate of drought-exposed oak shoots was slowed down or even stopped with increasing drought stress during the course of the growing season, as indicated by decreasing leaf water potentials [34]. This effect mainly occurred in 2009 when drought stress was strongest. Hence, not all oaks formed a 2^nd^ flush under drought conditions and, if they did, the onset was delayed until soil-water conditions became non-limiting. As discussed by [51] and [52], such a reduction of shoot phenological development may be a successful strategy for limiting leaf area and therefore transpirational water loss. Remarkably, the trees quickly recovered after re-watering and shoot growth was resumed. This recovery was in close synchrony with the fast up-regulation of photosynthetic activity [34] and increased evapotranspiration [27]. A similar recovery was also shown in a study with *Q. robur* seedlings, in that shoot growth of second and third flushes after re-watering was even stronger under drought than under control conditions [25]. These findings demonstrate the superior drought resistance and resilience of oaks, even during their most vulnerable stage of early growth, and confirm their high phenotypic plasticity [17]. It is likely that canopy trees will be able to cope even better with severe drought as they are able to tap into deeper water reservoirs with their long taproots [53], [54]. Also, transpiration might be reduced with increasing atmospheric CO_2_ concentrations, allowing soil moisture to sustain oak growth for an even longer period of time [55], [56].

## Conclusions

In conclusion, our study demonstrates advanced bud burst and earlier onset of intra-annual shoot growth as specific responses to air warming. However, these acclimatisation mechanisms do not necessarily lead to prolonged seasonal growth, higher biomass or increased carbon sequestration due to ontogenetically fixed development and/or limited availability of nutrient and soil water. With respect to drought, we found that oaks are able to cope well with reduced soil water availability, both in terms of resistance and resilience, by delaying or suspending individual shoot growth during the season. Furthermore, we found that bud burst is advanced by a carry-over effect from the previous year’s drought. We therefore strongly suggest further investigation on the nature of potential carry-over effects that might bias warming effects on trees under future climate scenarios.

## Supporting Information

Figure S1 Relationship of mean April air temperature in 2008 to bud burst phenology. Air temperature (°C, 0∶00 to 23∶00, UTC+1) and the day of the year, when 50% of the buds were open, are separately shown for each model ecosystem chamber. Data of the two soils and the 12 provenances were pooled because air temperature was equal within a chamber.
(TIF)

Figure S2 Bud burst development and total duration of shoot growth separately shown for all provenances. A: Average day of the year (2007 to 2009) when 50% of a tree’s buds were open (*n* = 16). B: average total duration of shoot growth in days (sum over all flushes, *n* = 8), separately shown for all treatments, the two soils (acidic and calcareous) and the 12 provenances, nested in 3 species (Qro: *Quercus robur*, T = Tägerwilen, B = Bonfol, H = Hühnenberg, Mi = Magadino; Qpe: *Q. petraea*, C = Corcelles, M = Magden, W = Wädenswil, G = Gordevio; Qpu: *Q. pubescens*, Lk = Leuk, LL = Le Landeron, P = Promontogno, A = Arezzo/Italy; a map of the provenance’s sites is presented by [17], climate conditions at the provenance’s sites are shown in detail by [19]). Asterisks indicate that a provenance (mean of CO and AW) is different from the mean of all other provenances (* = *P*<0.05, ** = *P*<0.01, *** = *P*<0.001). Further results of statistical analyses are given in Table 3.
(TIF)

Table S1 Correlation matrix between phenology and foliage biomass and stem diameter. Correlation (Pearson correlation coefficients) between bud burst, duration of growth per flush (period_1_: 1^st^ flush, period_2_: 2^nd^ flush, period_3_: 3^rd^ flush), total duration of growth (period_tot_), shoot length per flush (length_1_: 1^st^ flush, length_2_: 2^nd^ flush, length_3_: 3^rd^ flush), total shoot length per growing season growth (length_tot_), number of flushes (# flushes) and foliage biomass and stem diameter (Ø stem). Each parameter *n* = 2304).
(DOCX)
