# Supplementary figures and images for: A Phenological Timetable of Oak Growth under Experimental Drought and Air Warming
Source: PLoS One. 2014 Feb 24;9(2):e89724. doi: 10.1371/journal.pone.0089724 (PMC3933646; doi:10.1371/journal.pone.0089724)

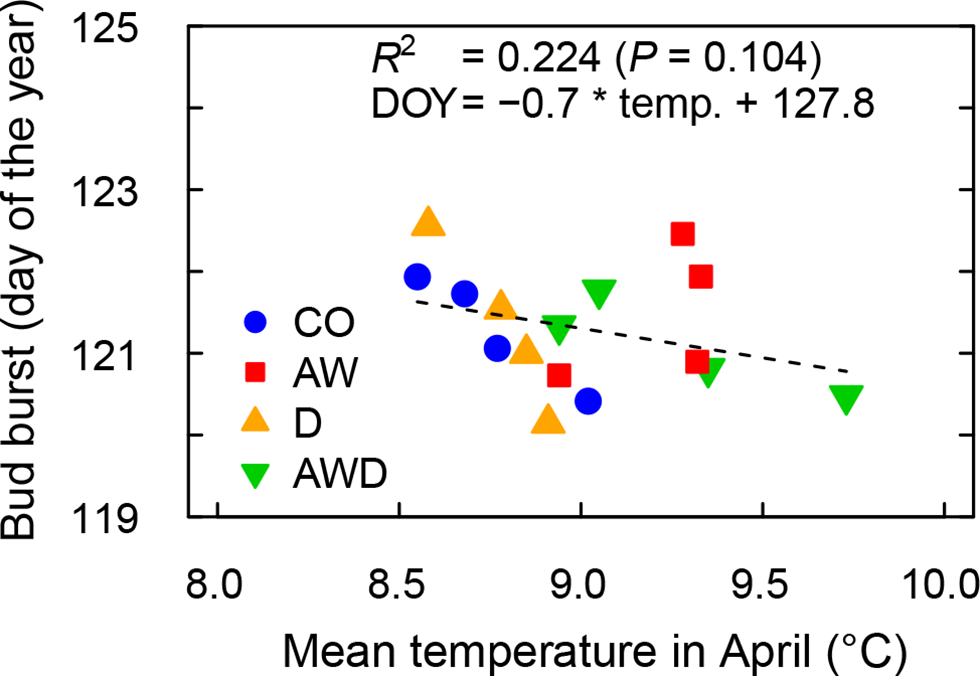

Supplement: Figure S1 — Relationship of mean April air temperature in 2008 to bud burst phenology. Air temperature (°C, 0∶00 to 23∶00, UTC+1) and the day of the year, when 50% of the buds were open, are separately shown for each model ecosystem chamber. Data of the two soils and the 12 provenances were pooled because air temperature was equal within a chamber. (TIF) [file pone.0089724.s001.tif]

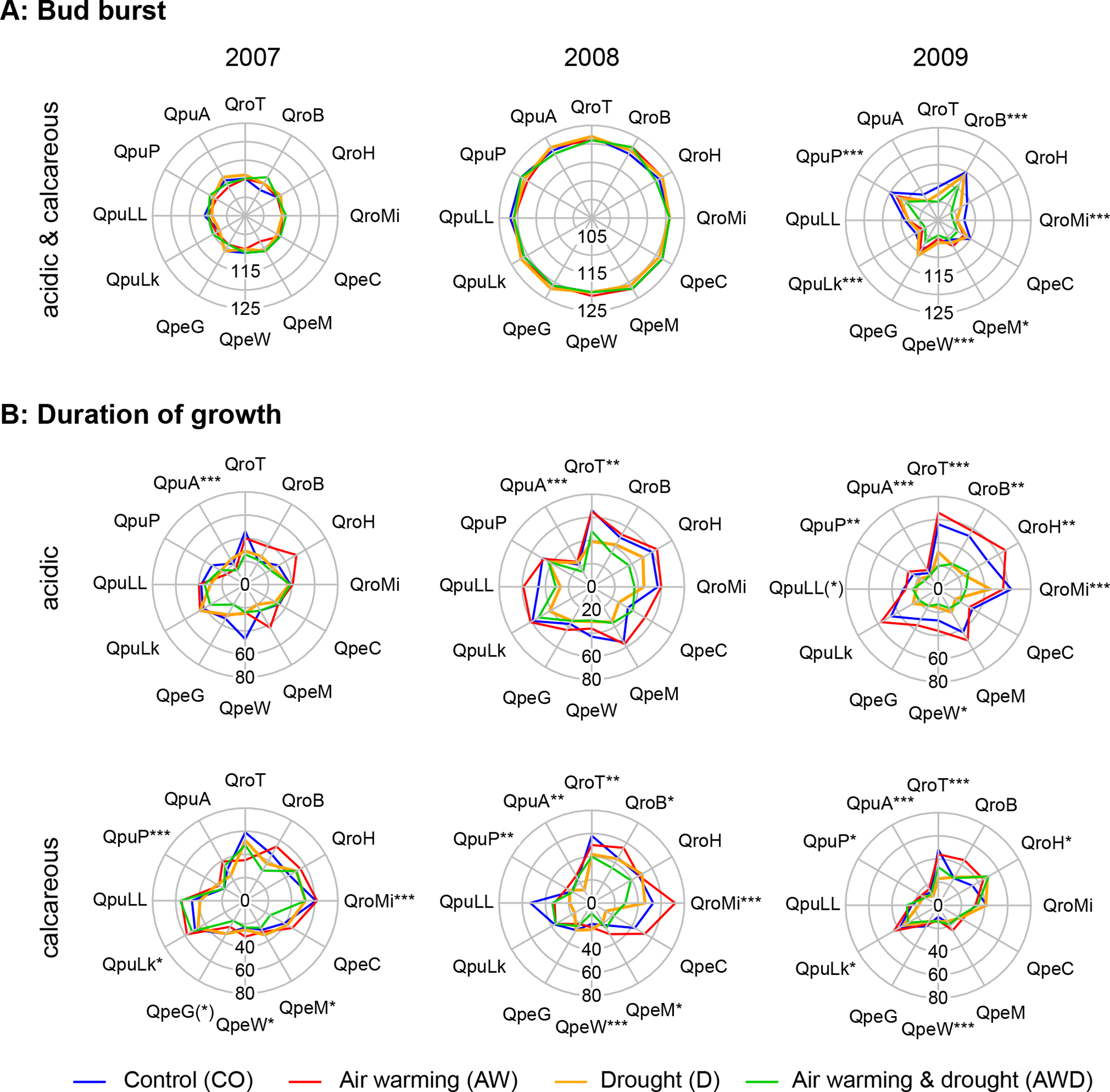

Supplement: Figure S2 — Bud burst development and total duration of shoot growth separately shown for all provenances. A: Average day of the year (2007 to 2009) when 50% of a tree’s buds were open (n = 16). B: average total duration of shoot growth in days (sum over all flushes, n = 8), separately shown for all treatments, the two soils (acidic and calcareous) and the 12 provenances, nested in 3 species (Qro: Quercus robur, T = Tägerwilen, B = Bonfol, H = Hühnenberg, Mi = Magadino; Qpe: Q. petraea, C = Corcelles, M = Magden, W = Wädenswil, G = Gordevio; Qpu: Q. pubescens, Lk = Leuk, LL = Le Landeron, P = Promontogno, A = Arezzo/Italy; a map of the provenance’s sites is presented by [17], climate conditions at the provenance’s sites are shown in detail by [19]). Asterisks indicate that a provenance (mean of CO and AW) is different from the mean of all other provenances (* = P<0.05, ** = P<0.01, *** = P<0.001). Further results of statistical analyses are given in Table 3. (TIF) [file pone.0089724.s002.tif]
