# Supplementary material for: A Phenological Timetable of Oak Growth under Experimental Drought and Air Warming
Source: PLoS One. 2014 Feb 24;9(2):e89724. doi: 10.1371/journal.pone.0089724 (PMC3933646; doi:10.1371/journal.pone.0089724)
Supplement: Table S1 — Correlation matrix between phenology and foliage biomass and stem diameter. Correlation (Pearson correlation coefficients) between bud burst, duration of growth per flush (period1: 1st flush, period2: 2nd flush, period3: 3rd flush), total duration of growth (periodtot), shoot length per flush (length1: 1st flush, length2: 2nd flush, length3: 3rd flush), total shoot length per growing season growth (lengthtot), number of flushes (# flushes) and foliage biomass and stem diameter (Ø stem). Each parameter n = 2304). (DOCX) [file pone.0089724.s003.docx]

**Table S1.** **Correlation matrix between phenology and foliage biomass and stem diameter.** Correlation (Pearson correlation coefficients) between bud burst, duration of growth per flush (period_1_: 1^st^ flush, period_2_: 2^nd^ flush, period_3_: 3^rd^ flush), total duration of growth (period_tot_), shoot length per flush (length_1_: 1^st^ flush, length_2_: 2^nd^ flush, length_3_: 3^rd^ flush), total shoot length per growing season growth (length_tot_), number of flushes (# flushes) and foliage biomass and stem diameter (Ø stem). Each parameter *n* = 2304).

|  | period_1_ | period_2_ | period_3_ | period_tot_ | length_1_ | length_2_ | length_3_ | length_tot_ | #flushes | foliage | Ø stem |
| --- | --- | --- | --- | --- | --- | --- | --- | --- | --- | --- | --- |
| bud burst | -0.056 | 0.202 | -0.191 | 0.131 | 0.167 | 0.234 | -0.049 | 0.190 | 0.118 | 0.077 | 0.040 |
| period_1_ |  | 0.231 | -0.237 | 0.237 | 0.603 | -0.069 | -0.198 | 0.110 | -0.217 | 0.461 | 0.547 |
| period_2_ |  |  | -0.077 | 0.689 | 0.324 | 0.533 | 0.010 | 0.451 | 0.006 | 0.263 | 0.409 |
| period_3_ |  |  |  | 0.626 | -0.199 | -0.023 | 0.332 | 0.101 | 0.044 | -0.134 | -0.143 |
| period_tot_ |  |  |  |  | 0.245 | 0.624 | 0.496 | 0.722 | 0.687 | 0.282 | 0.399 |
| length_1_ |  |  |  |  |  | 0.178 | -0.119 | 0.456 | -0.085 | 0.472 | 0.600 |
| length_2_ |  |  |  |  |  |  | 0.342 | 0.850 | 0.560 | 0.209 | 0.308 |
| length_3_ |  |  |  |  |  |  |  | 0.613 | 0.649 | 0.011 | 0.021 |
| length_tot_ |  |  |  |  |  |  |  |  | 0.622 | 0.325 | 0.443 |
| #flushes |  |  |  |  |  |  |  |  |  | 0.040 | 0.081 |
| foliage |  |  |  |  |  |  |  |  |  |  | 0.702 |
